# Ultrasonography is a valuable non-invasive tool for determining extravascular lung water in severe sepsis

**DOI:** 10.1186/cc9561

**Published:** 2011-03-11

**Authors:** M Kok, H Endeman

**Affiliations:** 1 Diakonessenhuis Utrecht, the Netherlands; 2 Onze Lieve Vrouwe Gasthuis, Amsterdam, the Netherlands

## Introduction

The aim of this study was to evaluate the value of ultrasonography of the lung in order to determine the level of volume load defined as the extravascular lung water index (ELWI). Hereto the presence of bilateral interstitial syndrome found by pulmonary ultrasonography is compared with ELWI as measured by thermodilution with PiCCO technology.

## Methods

A prospective study was carried out. The study was performed in the ICU of a medium-sized teaching hospital. Adult patients who were suffering from severe sepsis were included in the study. Ultrasonography (OptiGo; Philips) of the lung was categorized as an A-profile in cases of no signs of interstitial syndrome and a B-profile in cases of interstitial syndrome. Ultrasonography of both sides of the lung was performed. Therefore, the following profiles were determined: AA, AB and BB. The BB-profile, bilateral interstitial syndrome, is regarded as being associated with volume overload [1]. The ELWI was calculated after thermodilution by PiCCO technology in all patients and compared between the three different ultrasonographic profiles. Statistical analysis was performed by independent-sample *t *test. *P *< 0.05 was considered statistically significant.

## Results

**Figure 1:**
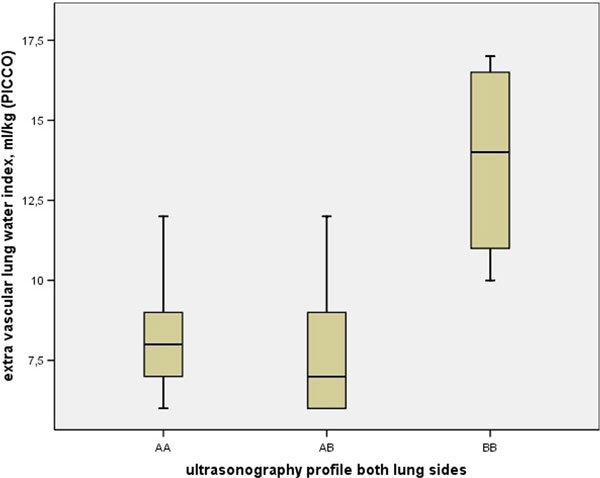
**Boxplots of the relationship between extravascular lung water (ELW, ml/kg) and the different profiles determined by ultrasonography of both lung sides, in patients with severe sepsis**.

In 11 consecutive patients (six men), ultrasonography of the lung was performed 27 times. Mean age was 70 years (SD 3.4), mean APACHE IV score 88 (SD 23) and APACHE II score 26 (SD 6). Most frequent reasons for ICU admission were sepsis, respiratory and renal failure. Mean ELWI in patients with the AA-profile (48.1% of the profiles) and the BB-profile (29.6%) was respectively 8.5 (SD 1.7) and 13.8 (SD 2.9). This difference was significant (*P *= 0.001). Mean ELWI of the AB-profile (22.2%) was 7.8.(SD 2.3). The mean ELWI of this profile also differed significantly with the BB-profile (*P *= 0.002). See Figure 1.

## Conclusions

Our study demonstrates the potential of ultrasonography in the detection of extravascular lung water in adult intensive care patients, suffering from severe sepsis. Since ultrasonography is an inexpensive, non-invasive and effective modality, the small study supports the use of ultrasonography as a possible tool in the evaluation of volume status in patients with severe sepsis. Larger studies are necessary to confirm these findings.
